# A 3-year study of *Candida* infections among patients with malignancy: etiologic agents and antifungal susceptibility profile

**DOI:** 10.3389/fcimb.2023.1152552

**Published:** 2023-05-12

**Authors:** Mahdieh Sharifi, Parisa Badiee, Mahdi Abastabar, Hamid Morovati, Iman Haghani, Mahta Noorbakhsh, Rasoul Mohammadi

**Affiliations:** ^1^ Department of Medical Parasitology and Mycology, School of Medicine, Isfahan University of Medical Sciences, Isfahan, Iran; ^2^ Clinical Microbiology Research Center, Shiraz University of Medical Sciences, Shiraz, Iran; ^3^ Invasive Fungi Research Center, Mazandaran University of Medical Sciences, Sari, Iran; ^4^ Department of Medical Mycology, School of Medicine, Mazandaran University of Medical Sciences, Sari, Iran; ^5^ Department of Parasitology and Mycology, School of Medicine, Shiraz University of Medical Sciences, Shiraz, Iran; ^6^ Department of Infectious Diseases, School of Medicine, Isfahan University of Medical Sciences, Isfahan, Iran; ^7^ Infectious Diseases and Tropical Medicine Research Center, Isfahan University of Medical Sciences, Isfahan, Iran

**Keywords:** *Candida* infections, malignancy, antifungal susceptibility, molecular identification, epidemiology

## Abstract

**Objective:**

Opportunistic fungal infections by *Candida* species arise among cancer patients due to the weakened immune system following extensive chemotherapy. Prophylaxis with antifungal agents have developed the resistance of *Candida* spp. to antifungals. Accurate identification of yeasts and susceptibility patterns are main concerns that can directly effect on the treatment of patients.

**Methods:**

Over a period of three years, 325 cancer patients suspected to *Candida* infections were included in the current investigation. The clinical isolates were molecularly identified by polymerase chain reaction-restriction fragment length polymorphism (PCR-RFLP). All strains, were examined for *in vitro* susceptibility to the amphotericin B, itraconazole, fluconazole, and anidulafungin according to the CLSI M27 document.

**Results:**

Seventy-four cancer patients had *Candida* infections (22.7%). *Candida albicans* was the most common species (83.8%). Antifungal susceptibility results indicated that 100% of the *Candida* isolates were sensitive to amphotericin B; however, 17.6%, 9.4%, and 5.4% of clinical isolates were resistant to anidulafungin, fluconazole, and itraconazole, respectively.

**Conclusion:**

The findings of the present work shows a warning increase in resistance to echinocandins. Since all fluconazole resistance isolates were obtained from candidemia, we recommend amphotericin B as the first line therapy for this potentially fatal infection.

## Introduction

Candidiasis is an opportunistic fungal infection closely connected to malignancies and the complications of their treatment. Incidence of *Candida* infections has been showed to be ranging from 7 to 52% among cancer patients (Lone et al., 2014). The cytotoxic anti-cancer drugs have severe effects on mucosal immune defense, leading to *Candida* colonization. *Candida* may impel some types of cancer including oral squamous cell carcinoma (OSCC) by using of carcinogenic compounds production such as nitrosamines and N−nitrosobenzylmethylamine (Krogh et al., 1987). Although *C. albicans* is the most prevalent species, but non-*albicans Candida* species including *C. tropicalis*, *Pichia kudriavzevii* (*C. krusei*), *Meyerozyma guilliermondii* (*C. guilliermondii*), *C. glabrata, C. parapsilosis*, and *Kluyveromyces marxianus* (*C. kefyr*) with a reduced susceptibility to echinocandins and triazoles become a consequential clinical challenge (Eddouzi et al., 2013). Prophylaxis with azoles and echinocandins among vulnerable populations, have been connected to a shift from *C*. *albicans* to non-*albicans Candida* species in some countries (Cleveland et al., 2012; Pfaller et al., 2014a). Since there are main differences in species distributions and drug susceptibilities in various regions, we aimed to determine *Candida* distribution and antifungal susceptibility of clinical isolates among cancer patients in Isfahan, Iran.

## Materials and methods

### Patients

A total of 325 suspected cases referred to 4 university hospitals (Al-Zahra, Seyed-al-Shohada, Imam Hossein, and Amin) were included in this study from April 2018 to June 2021 based on clinical manifestations. Patients who had not received haematological/oncological treatment within the past 12 months were excluded from the study. All types of *Candida* infections were included. Written informed consent for participation provided by all patients.

### Molecular identification

#### PCR-RFLP

Boiling method was used to extract genomic DNA (Silva et al., 2012). Briefly, a loopful of fresh colony was suspended in 80 µL of double distilled water and boiled for 20 min, then centrifuged for 8 min at 6000 rpm, and then the supernatant (containing DNA) was used for PCR. The ITS1-5.8S-ITS2 region was amplified using the universal primers ITS1 (5-TCCGTAGGTGAACCTGCGG-3) and ITS4 (5- TCCTCCGCTTATTGATATGC-3) (White et al., 1990) in a final volume of 25 μl, containing 2 μl of extracted DNA, 0.4 mM of dNTPs, 1.5 mM of MgCl_2_, 30 pmol of each primer, 1.25 U of Taq DNA polymerase, and 2.5 μl of 10× PCR buffer. The following program was set for PCR: 1 cycle at 95°C for 5 min, followed by 30 cycles of 1 min at 94°C, 45 sec at 55°C, and 45 sec at 72°C, with a final extension step at 72°C for 7 min. Digestion of PCR products were performed with 1U of restriction enzyme *Msp*I (Fermentas, Vilnius, Lithuania) in a final reaction volume of 15 μl containing 3.5 μl water, 1.5 μl buffer, and 10 μl PCR product at 37°C for 20 min (FastDigest™). PCR amplicons and RFLP products were run onto 1.5% and 2% agarose gel electrophoresis, respectively. The products were stained with SYBR Safe DNA gel stain (1:10,000 dilution in Tris/Borate/EDTA) and then photographed.

#### Antifungal susceptibility testing

According to the clinical and laboratory standard institute (CLSI) document M27 (Clinical and Laboratory Standards Institute (M27), 2017), minimum inhibitory concentrations (MICs) of antifungals viz. fluconazole (Sigma-Aldrich, Germany), amphotericin B (Sigma-Aldrich, Germany), itraconazole (Janssen Research Foundation, Beerse, Belgium), and anidulafungin (Cayman Chemical, USA) were assessed. Final concentrations for antifungal agents were as follows: itraconazole and amphotericin B (0.0313–16 μg/ml), fluconazole (0.125–64 μg/ml), and anidulafungin (0.015–8 μg/ml). A serial dilution of each antifungal was prepared in RPMI1640 medium (with L-Glutamine, without bicarbonate) (Sigma Chemical Co., St. Louis, MO, USA). Compared to a McFarland standard; no. 0.5 = 1–5×10^6^ CFU/ml, the desired final inoculum size in the wells was 0.5×10^3^ to 2.5×10^3^ CFU/ml after 100 μl inoculation. Microplates were incubated at 35°C, and the MICs were visually determined after 24 h. The MIC endpoint for fluconazole, itraconazole, and anidulafungin have been described as the level which inhibited a significant growth of fungus (50%) compared to drug-free growth control, while for amphotericin B, 100% growth inhibition is considered. **Table 1** shows interpretive breakpoints for *in vitro* susceptibility testing of *Candida* species according to M27 and M60 documents, Borman et al., and Mroczyńska, et al. (Clinical and Laboratory Standards Institute (M27), 2017; Clinical and Laboratory Standards Institute (M60), 2017; Borman et al., 2020; Mroczyńska and Brillowska-Dąbrowska, 2020).

**Table 1:** Interpretive guidelines for antifungal susceptibility testing of *Candida* species.

Antifungal agent	*Candida* species	Breakpoints (μg/mL)		
		S	SDD (OR) I	R
Amphotericin B	*C. albicans* *C. glabrata* *C. tropicalis* *C. parapsilosis* ^a^ *C. krusei* ^b^ *C. famata*	<1 <1 <1 <1 <1 N/A	N/A N/A N/A N/A N/A N/A	≥1 ≥1 ≥1 ≥1 ≥1 N/A
Fluconazole	*C. albicans* *C. glabrata* *C. tropicalis* *C. parapsilosis* *C. krusei* *C. famata*	≤2 N/A ≤2 ≤2 N/A N/A	4 ≤32 4 4 N/A N/A	≥8 ≥64 ≥8 ≥8 N/A N/A
Itraconazole	*C. albicans* *C. glabrata* *C. tropicalis* *C. parapsilosis* *C. krusei* *C. famata*	≤0.125 ≤0.125 ≤0.125 ≤0.125 ≤0.125 N/A	0.25-0.5 0.25-0.5 0.25-0.5 0.25-0.5 0.25-0.5 N/A	≥1 ≥1 ≥1 ≥1 ≥1 N/A
Anidulafungin	*C. albicans* *C. glabrata* *C. tropicalis* *C. parapsilosis* *C. krusei* *C. famata*	≤0.25 ≤0.12 ≤0.25 ≤2 ≤0.25 N/A	0.5 ≤0.25 0.5 4 0.5 N/A	≥1 ≥0.5 ≥1 ≥8 ≥1 N/A

^^a^^The breakpoints of fluconazole has not been described for C. krusei, because it is intrinsically resistant to fluconazole; ^b^Antifungal agents have no breakpoints for uncommon *Candida* species such as *C. famata*. S, Susceptible; I, Intermediate; R, Resistant; and SDD, Susceptible dose dependent; N/A, Not applicable.

### Statistical analysis

The relationship among *Candida* infections and age of patients, gender, and type of cancer was adjusted using Fisher’s exact test and Mann–Whitney U−test. A *P*-value less than 0.05 was considered statistically significant. The MIC range, MIC_50_ (the minimum concentration of antifungal agent at which 50% of isolates are inhibited) and MIC_90_ (the minimum concentration of antifungal agent at which 90% of isolates are inhibited) were determined.

## Results

**Figure 1:**
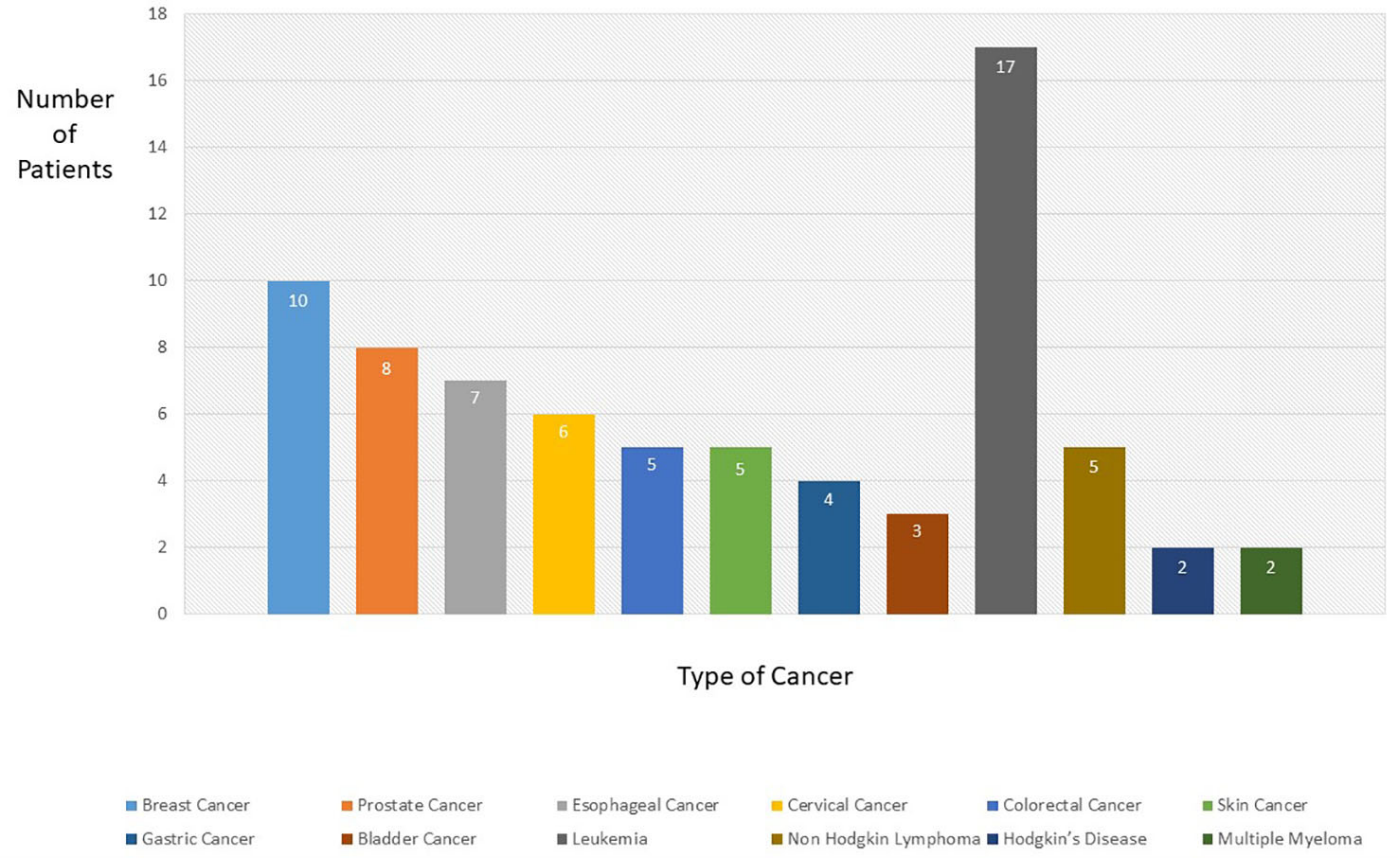
Classification of patients according to the type of cancer.

**Figure 2:**
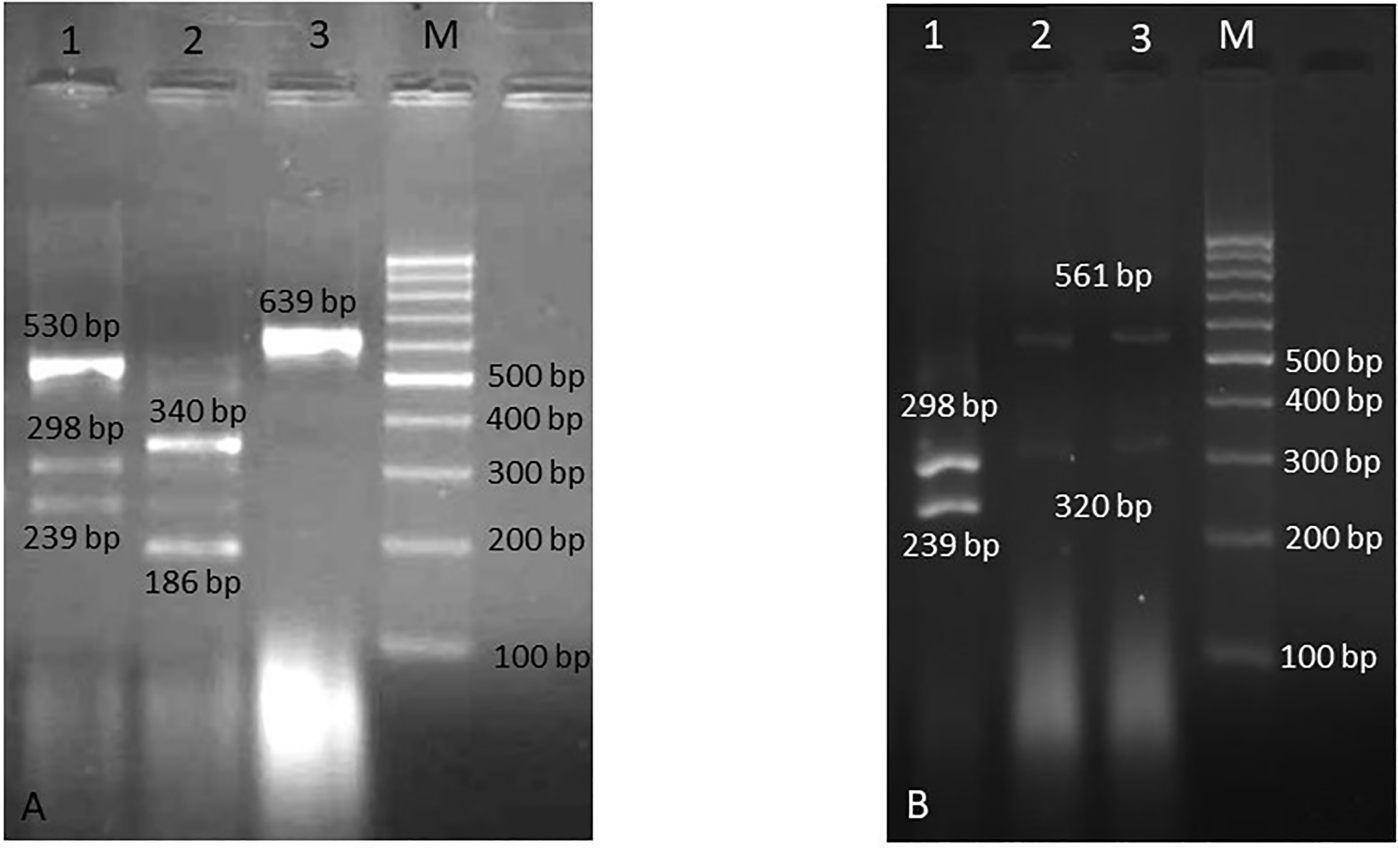
Agarose gel electrophoresis of RFLP products. **(A)** Lane 1: *C. albicans* and *C. parapsilosis* (mixed), lane 2: *C. tropicalis*, lane 3: *C. famata*, and M is 100 bp DNA size marker, **(B)** Lane 1: *C. albicans*, lanes 2, 3: *C. glabrata*, and M is 100 bp DNA size marker.

Over the investigation period, 74 cases of *Candida* infection were detected. Forty-eight patients (64.8%) had solid organ tumors and 26 patients (35.2%) had haematological malignancies (**Figure 1**). Age range was between 1-89 years with median age of 47.3. Gender ratio was 43 males per 31females. The most *Candida* species were isolated from urine (n=24), bronchoalveolar lavage (BAL) (n=15), blood (n=13), and esophageal biopsies (n=7) (**Table 2**). *Candida albicans* was the most prevalent species (83.8%) followed by *C. glabrata* (5.4%), *C. tropicalis* (4%), *C. parapsilosis* (4%), *C. krusei* (1.3%), and *C. famata* (1.3%) (**Figure 2**; **Table 2**). The prevalence of *C. albicans* was significantly higher than other species (*P* = 0.029). In solid organ tumor group, the type of cancer was not statistically different (*P* = 0.085); however, in patients with haematological malignancies, leukemia was significantly more common among patients (*P* = 0.043) (**Table 3**). Antifungal susceptibility results are shown in **Table 4**. Briefly, our findings indicated that all clinical isolates (100%) were sensitive to amphotericin B. The lowest activity was observed in anidulafungin against *Candida* spp. In addition, 83.8% and 81.1% of isolates were sensitive to fluconazole and itraconazole, respectively.

**Table 2:** Clinical specimens from which *Candida* species were isolated.

Clinical Specimens	*Candida* species	Total Number (%)
Urine	*C. albicans* (n=19), *C. glabrata* (n=1), *C. tropicalis* (n=1), *C. parapsilosis* (n=1), *C. krusei* (n=1), *C. famata* (n=1)	24 (32.4%)
BAL	*C. albicans* (n=13), *C. glabrata* (n=1), *C. parapsilosis* (n=1)	15 (20.3%)
Blood	*C. albicans* (n=12), *C. glabrata* (n=1)	13 (17.5%)
Esophageal Biopsy	*C. albicans* (n=5), *C. tropicalis* (n=2)	7 (9.4%)
Gastric Biopsy	*C. albicans* (n=4)	4 (5.4%)
Wound	*C. albicans* (n=3), *C. parapsilosis* (n=1)	4 (5.4%)
Thrush	*C. albicans* (n=2)	2 (2.7%)
Ascites	*C. albicans* (n=2)	2 (2.7%)
Perleche	*C. albicans* (n=1)	1 (1.3%)
Abscess	*C. albicans* (n=1)	1 (1.3%)
Nail	*C. glabrata* (n=1)	1 (1.3%)
Total	*C. albicans* (n=62), *C. glabrata* (n=4), *C. tropicalis* (n=3), *C. parapsilosis* (n=3), *C. krusei* (n=1), *C. famata* (n=1)	74 (100%)

**Table 3:** Statistical analysis for *Candida* infections and variables of age, gender and type of cancer.

Variable	Age	Gender	Type of Cancer	
			Solid Organ Tumors	Haematological Malignancies
*P*-value (SD/NSD)				
***Candida* Infections**	*P* = 0.67 (NSD)	*P* = 0.15 (NSD)	*P* = 0.085 (NSD)	*P* = 0.043 (SD)

SD, Significant difference; NSD, No significant difference.

**Table 4:** MIC range, MIC_50_, MIC_90_, and geometric mean of the antifungals against *Candida* spp. and susceptibility pattern of clinical isolates.

*Candida *spp.	MIC range (μg/mL)	MIC_50_ (μg/mL)	MIC_90_ (μg/mL)	GM	S	I/SDD	R
					Number of Isolates		
***C. albicans***	AmB (0.0313-0.5) FLZ (0.125-64) ITZ (0.0313-4) AFG (0.015-1.92)	0.125 1 0.062 0.12	0.5 4 0.25 0.48	0.141 0.892 0.078 0.106	62 53 53 42	0 3 6 14	0 6 3 6
***C. glabrata***	AmB (0.0313-0.5) FLZ (0.5-32) ITZ (0.0313-4) AFG (0.015-4)	0.25 0.5 0.0313 0.015	0.5 32 4 4	0.176 1.68 0.148 0.173	4 3 3 2	0 0 0 0	0 1 1 2
***C. tropicalis***	AmB (0.0313-0.5) FLZ (1-2) ITZ (0.125-0.5) AFG (0.5-2)	0.062 2 0.25 1	0.5 2 0.5 2	0.099 1.58 0.25 1	3 3 1 0	0 0 2 0	0 0 0 3
***C. parapsilosis***	AmB (0.0313-0.5) FLZ (0.25-2) ITZ (0.0313-0.062) AFG (0.015-2)	0.125 0.5 0.0313 0.03	0.5 2 0.062 2	0.125 0.629 0.039 0.096	3 3 3 2	0 0 0 0	0 0 0 1
***C. krusei***	AmB (0.5) FLZ (4) ITZ (0.25) AFG (4)	N/A N/A N/A N/A	N/A N/A N/A N/A	N/A N/A N/A N/A	1 N/A 0 0	0 N/A 1 0	0 N/A 0 1
^a^ *C. famata*	AmB (0.5) FLZ (64) ITZ (16) AFG (8)	N/A N/A N/A N/A	N/A N/A N/A N/A	N/A N/A N/A N/A	N/A N/A N/A N/A	N/A N/A N/A N/A	N/A N/A N/A N/A

a Antifungal agents have no breakpoints for uncommon *Candida* species such as *C. famata*. GM, geometric mean; S, susceptible; I, intermediate; R, resistant; and SDD, susceptible dose dependent; AmB, amphotericin B; FLZ, fluconazole; ITZ, itraconazole; AFG, anidulafungin; N/A, Not applicable.

## Discussion

*Candida* infections can be considered as an important sign of immunosuppression in patients with malignancies. Most cancer patients are neutropenic (diminution of blood neutrophils to less than 1500/mm^3^) due to the cytotoxic chemotherapy, hematological disorders, and acute leukemia (Walsh and Gamaletsou, 2013). This condition is one of the main predisposing factors for invasive fungal infections (IFIs) such as invasive aspergillosis and candidiasis (Spiess et al., 2007). The incidence of fungal infections among cancer patients is connected to the immune system status, antifungal resistance rates, and type of malignancy (Bhatt et al., 2011). In the present study, the most clinical samples were obtained from urine (32.4%). Candiduria in cancer patients should be considered as a marker for disseminated candidiasis and appropriate antifungal therapy is needed (Georgiadou et al., 2013). All patients with candiduria were treated with fluconazole; however, in three patients (12.5%), amphotericin B were added to their regimen due to the positive blood culture. The presence of *Candida* in the upper respiratory tract of cancer patients is usual, and cannot be evaluated as invasive pulmonary *Candida* infection. Only histopathological examinations can prove the infection (Kontoyiannis et al., 2002), nevertheless, none of the patients with pulmonary symptoms (n=15) underwent pathology examination, and this is one of the main limitations of our study. Candidemia is a fatal fungal infection nearly related to cancer and the difficulties of its treatment. Here, we isolated 13 *Candida* strains (17.5%) from patients with bloodstream infections (BSIs). Interestingly, except for one case, all BSIs were caused by C*. albicans*, and all fluconazole-resistant isolates were obtained from the blood samples. Gastroesophageal biopsies were in the fourth place of clinical specimens that were collected endoscopically. Gastroesophageal candidiasis have been shown to be increasing in cancer patients who consuming acid suppressing therapy (AST) and proton pump inhibitors (PPIs) (Daniell, 2016), because these drugs change the gastric pH, which can encourage *Candida* colonization of the esophagus (patients with reflux) and gastrointestinal tract (Shah et al., 2015). In agreement, all patients were taking PPIs (omeprazole; n=6, pantoprazole; n=3, and lansoprazole; n=2) in this investigation. Although stomach and esophageal cancers were the most prevalent malignancies reported by Kolahdoozan et al. in 2010 (Kolahdoozan et al., 2010); however, leukemia and breast cancers were the most common types of cancers in our study (**Figure 1**). Nine out of 13 patients with candidemia had leukemia (69.2%). *Candida albicans* is the most common opportunistic yeast in the clinical setting, which causes a widespread of infections ranging from mucocutaneous lesions to lethal deep-tissue infections (Pu et al., 2017). We also revealed *C. albicans* as the most frequent species in this survey (83.8%) which was isolated from all specimens except nail infection. *In vitro* antifungal activities of four antifungal agents were assessed for clinical isolates, and we found that 17.6% of *Candida* spp. were resistant to anidulafungin, 9.4% to fluconazole, and 5.4% to itraconazole. None of them were resistant to amphotericin B. Our results are in line with Hamzehee et al. (2019) which reported a 9.52% and 4.7% resistance to fluconazole and itraconazole, respectively. Unlike the study of Sharifynia et al. (2016), who reported 62.5% resistance to amphotericin B, in the present study, all isolates were sensitive to amphotericin B, which were consistent with the results of the Roy study (Roy et al., 2013). Infectious Diseases Society of America (IDSA) proposes the echinocandins (micafungin, caspofungin, and anidulafungin) as the newest class of antifungals for disseminated *Candida* infections as initial therapy in both neutropenic and non-neutropenic patients (Pappas et al., 2016), nevertheless, fluconazole is still broadly consumed due to its availability for both parenteral and enteral administration and low cost of the drug. Our findings showed that resistance to anidulafungin was more than fluconazole, and 6 out of 12 resistant isolates (50%) were obtained from candidemia. Unlike the study of Mohamed et al. (2018), which the most common strains were non-*albicans* (70.7%), in the present study, 83.8% of the clinical isolates were *C. albicans*. This variation in different geographical areas is related to many factors such as patient demographic features, various antifungal therapy practices, chronic underlying diseases, and use of indwelling catheters (Yapar, 2014). *Candida tropicalis* is main reported non-*albicans Candida* species in Asia and tropical regions (Tan et al., 2016) compared to the United States and Europe, where *C. glabrata* is the most leading non-*albicans* species (Falagas et al., 2010); however, *C. glabrata* was the most prevalent non-*albicans Candida* species in the current study. Increased number of *C. glabrata* isolates in Iran (Aslani et al., 2018; Mardani et al., 2020), may be related to the overuse of fluconazole as prophylaxis and treatment.

## Conclusion

Although prophylaxis with azoles and echinocandins is one of the main factors in shifting the etiologic agents from *albicans* to non-*albicans* (Pfaller et al., 2014a), but our results revealed that *C. albicans* was still the leading cause of infection. The echinocandin anidulafungin is broadly used as first-line antifungal therapy for invasive candidiasis and candidemia (Pfaller et al., 2014b); nevertheless, the antifungal resistance was predominantly restricted to anidulafungin (17.6%) and fluconazole (9.4%) in the present study. Among non-*albicans Candida* species, all *C. tropicalis* were resistant to anidulafungin, and *C. glabrata* showed the most resistance to antifungal agents. Since the resistance to echinocandins and other antifungal agents is still infrequent, it alarms for an uninterrupted inspection and exacerbation of antifungal stewardship policies to decrease acquired resistance among clinical isolates.

## Data availability statement

The original contributions presented in the study are included in the article/supplementary material. Further inquiries can be directed to the corresponding author.

## Ethics statement

This research was approved by the Ethics committee of Isfahan University of Medical Sciences (no. IR.MUI.MED.REC.1399.1056). Written informed consent to participate in this study was provided by the participants’ legal guardian/next of kin.

## Author contributions

Writing original draft preparation: RM. Patient’s follow-up and data collection: MS, MN and RM. Identification of the fungi: RM, MS and HM. Antifungal susceptibility testing: MS, PB, MA and IH. Reviewing and editing the manuscript: RM, HM and PB. All authors contributed to the article and approved the submitted version.
